# Carotid Intima-Media Thickness Is Predicted by Combined Eotaxin Levels and Severity of Hepatic Steatosis at Ultrasonography in Obese Patients with Nonalcoholic Fatty Liver Disease

**DOI:** 10.1371/journal.pone.0105610

**Published:** 2014-09-30

**Authors:** Giovanni Tarantino, Susan Costantini, Carmine Finelli, Francesca Capone, Eliana Guerriero, Nicolina La Sala, Saverio Gioia, Giuseppe Castello

**Affiliations:** 1 Department of Clinical Medicine and Surgery, Federico II University Medical School of Naples, Naples, Italy; 2 Centro Ricerche Oncologiche di Mercogliano, Istituto Nazionale Per Lo Studio E La Cura Dei Tumori “Fondazione Giovanni Pascale”, IRCCS, Mercogliano, Italy; 3 Center of Obesity and Eating Disorders, Stella Maris Mediterraneum Foundation, Chiaromonte, Potenza, Italy; University of Kansas Medical Center, United States of America

## Abstract

**Background:**

Non-Alcoholic Fatty Liver Disease (NAFLD) is a distinct coronary artery disease (CAD) risk factor. The atherosclerotic process predisposing to CAD includes altered lipid profile and inflammatory processes. The available evidence suggests that increased circulating levels of eotaxin, an eosinophil chemoattractant cytokine implicated in allergic responses, are detected in the serum of patients with CAD. Relationships were sought between serum eotaxin on the one hand, and intima-media thickness—an early predictor of the atherosclerotic process, hepatic steatosis, arterial blood pressure values, as well as inflammation/immune markers and angiogenetic factors—on the other.

**Methods:**

Eighty obese patients with NAFLD, diagnosed at ultrasonography, without evident cytolysis, formed our study population. Anthropometric measures, metabolic profile, serum concentrations of interleukin-1β, C-reactive protein, interleukin-6, fibrinogen, ferritin, TNF-α, spleen size, vascular endothelial growth factor, platelet-derived growth factor-BB and *heat shock* protein-70 were evaluated.

**Results:**

Serum eotaxin concentrations were distinctly associated with TNF α, IL-6, IL-1β, VEGF and PDGF-BB levels but not with CRP, fibrinogen, *heat shock* protein-70 or spleen size. Among the metabolic and anthropometric parameters, a significant predictive power emerged when comparing eotaxin to insulin resistance, expressed as HOMA. NAFLD was distinctly associated with HOMA (P = 0.0005). Intima-media thickness was well predicted by both eotaxin levels and severity of NAFLD at ultrasonography, although no relation was detected between these last two variables.

**Discussion and Conclusion:**

A role for insulin resistance in mediating the interplay between eotaxin and other inflammation/immune parameters could be evidenced in the induction/maintenance of atherosclerosis of obese patients with NAFLD.

## Introduction

Obesity and the Metabolic Syndrome (MS) contribute to early atherosclerosis, which is closely associated with Coronary Artery Disease (CAD). The basic atherosclerotic mechanisms include altered lipid profile and inflammatory processes [1], [2]. Recently, it has been evidenced that atherosclerosis, surprisingly found in pre-modern human beings, could be due to a basic predisposition rather than to contemporary lifestyles [3]. Mounting evidence continues to support a key role for immune/allergic mechanisms in various phases of atherosclerosis [4], as confirmed by an up-to-date research showing that IgE stimulates human and mouse arterial cell apoptosis as well as cytokine expression, and promotes atherogenesis in ApoE-/- mice [5]. Despite some criticisms [6], findings suggest that increased circulating levels of eotaxin, an eosinophil chemoattractant cytokine implicated in allergic responses, are detected in the serum of patients with CAD [7], [8]. Further observations have evidenced that the expression of Tenascin C controls eotaxin levels in apo E-/- mice, confirming its key role in the development of atherosclerosis [9]. The over-expression of eotaxin and the CCR3 receptor in human atherosclerosis has been highlighted also by genomic differential expression technology [10]. What is more, Eotaxin was positively associated with liver triglycerides in animal models [11]. It should be stressed that ectopic fat storage forms the pathological basis of the unclassified Non-Alcoholic Fatty Liver Disease (NAFLD), or non-Alcoholic Hepatic Steatosis (HS). The latter, well-detected by Ultrasound (US) [12], is closely associated with CAD [13], which has gained great relevance in recent years. US bears good sensitivity and specificity (92 and 100% respectively) for the histological diagnosis of NAFLD [14]. Obese people show increased Intramuscular TriGlycerides (ImTG) accumulation [15]; this fat deposition, which is due to an altered production of mitochondrial ATP, is linked to Insulin Resistance (IR), a mechanism central also to HS, and global CAD risk [16]. By increasing the number of reflections and thus, muscle echo intensity, ImTG can easily be evaluated by US [17]. Waist Circumference (WC) and Waist to Hip ratio (WHR) are strictly associated with CAD [18]. The concentration of High Density Lipoprotein-cholesterol (HDL) negatively predicts CAD [19]. Common carotid Intima-Media Thickness (IMT) is a sensitive method to detect early atherosclerosis [20].

Since there is still debate over the possible role of eotaxin in CAD, we tried to shed light on this controversy and elucidate some aspects of the complex mechanisms involved in the early atherosclerosis affecting NAFLD patients. Accordingly, relationships were sought between serum eotaxin and strict CAD risk factors, such as IMT, HS and arterial blood pressure. We further analyzed possible associations between eotaxin and j) anthropometric measures, jj) metabolic profile, jjj) HS grade at US and liver enzymes, jjjj) inflammation/immune markers, such as IL-1β, with its strictly related C-Reactive Protein (CRP) and Interleukin-6 (IL-6), fibrinogen, ferritin, Tumor Necrosis F-alpha (TNF-α), as well as Spleen Longitudinal Diameter (SLD) [21], jjjjj) angiogenetic factors—the former secreted by adipose tissue-derived stem cells, i.e., Vascular Endothelial Growth Factor (VEGF) [22] and the latter synthesized, stored and released by platelets upon activation, i.e., Platelet-Derived Growth Factor-BB (PDGF-BB) [23], both of which play an important role in vascular remodeling during atherosclerosis, and finally jjjjjj) circulating levels of the heat shock protein-70 (HSP-70)—a chaperone involved in inflammation, endoplasmic reticulum stress and apoptosis at liver and endothelial level [24].

## Patients and Methods

This cross-sectional study was carried out screening 125 consecutive, long-lasting obese individuals, referred to our out-patient metabolic unit from May 2012 to April 2013, who had been on a balanced low calorie, low fat (25% of calories) diet during the three months prior to enrolment and were characterized by sedentary life-style. The research protocols were approved by the Ethics Committee of the Federico II University Medical School of Naples (assigned protocol number: 231-05). All participants provided their written informed consent to participate in this study. The Department of Clinical Medicine and Surgery of the Federico II University Medical School of Naples (Italy) approved the conduction of this study.

Of the initial group of participants, 12 were excluded because they had problems performing abdominal US for NAFLD screening (intestinal meteorism), nine others because they had taken steroids (six for bronchial asthma, two for rheumatoid arthritis and one for IBD), and eight had undergone therapy with one or more drugs known to alter laboratory data, i.e., aspirin, statins, fibrates and metformin. Six patients were excluded for a history of hepatic co-morbidities (HBV/HCV infection or alcohol abuse). Ten patients were excluded for lack of adherence to the protocol.

Eighty obese patients with NAFLD formed the final population.

### Anthropometric and metabolic profile

The three degrees of obesity (light, moderate and severe) were established on the basis of BMI cut-off points of 30–34.9, 35–39.9 and>40 kg/m^2^, respectively. Visceral obesity was identified by measuring WC at the midpoint between the lower border of the rib cage and the iliac crest. Hip circumference was measured around the widest part of the buttocks, with the tape parallel to the floor, and Waist to Hip ratio (WHR) was calculated following the guide-lines for Europid reported in the “Waist Circumference and Waist–Hip Ratio: Report of a WHO Expert Consultation Geneva, 8–11 December 2008”. Accordingly, reference values of the waist circumference were set at 96 cm for men and 83 cm for women; WHR for general risk and obesity: 0.96 for men and 0.83 for women. IR status was determined by the HOmeostatic Metabolic Assessment (HOMA), which was assessed by the formula: fasting insulin (µU/mL) x fasting glucose (mg/dL)/405. Moreover, as the repeated HOMA measurements presented a high within-person variability in obese patients, HOMA values were averaged on the basis of at least five determinations to avoid misclassification.

### Criteria for diagnosing NAFLD

Obese patients, independently of evident hepatic cytolysis, were diagnosed as having NAFLD if they satisfied j) an inclusion criterion, i.e., the presence of hyper-echogenity, the so-called “bright liver”, based on a three–grade scale at US, as discussed below and jj) an exclusion criterion, i.e., the absence of any viral, autoimmune, metabolic disease, e.g. Wilson disease, hemochromatosis or anti-trypsin deficiency, which were ruled out by appropriate testing, following the generally accepted diagnostic guidelines. Furthermore, in case of hyper-transaminasemia, celiac disease was excluded estimating IgA anti-tissue trans-glutaminase antibodies. Alcohol abuse was screened according to DSM-IV diagnostic criteria and by means of the MAST (Michigam Alcohol Screening Test) and CAGE (Cut down, Annoyed, Guilty, Eye opener) tests, as well as by random tests for blood alcohol concentrations and the use of surrogate markers such as Mean Corpuscular Volume.

### Ultrasound evaluation

US measurements were performed operating on an Esaote system, i.e., Technos Partner (Genoa, Italy). Specifically, transverse scanning was performed to measure Subcutaneous Adipose Tissue (SAT) and Visceral Adipose Tissue (VAT) using an eleven linear probe and a 3.5 MHz convex probe, respectively. SAT was defined as the thickness between the skin-fat interface and the linea alba, avoiding compression. VAT was defined as the distance between the anterior wall of the aorta and the internal face of the recto-abdominal muscle perpendicular to the aorta, measured one cm above the umbilicus. When the aortic walls were not visualized, as when they were obscured by bowel gas, the Doppler scan was used. Spleen Longitudinal Diameter (SLD) was chosen to evaluate spleen volume and was carried out by postero-lateral scanning with a 3.5 MHz convex probe. Maximum length (the optically greatest overall longitudinal dimension obtained from one of the two poles) and cranio-caudal length (the optically maximal transversal dimension intercepting one of the two poles) were measured; the resulting values were then averaged, since the two measurements do not always coincide. The classification of HS, commonly known as “bright liver”, was based on the following scale of hyper-echogenity: grade 0 =  absent, grade 1 =  light, grade 2 =  moderate, grade 3 =  severe, indicating the difference between the densities of the liver and the right kidney. Technically, echo intensity can be influenced by many factors, particularly gain intensity. To avoid confounding factors that could modify echo intensity and thus bias comparisons, mean brightness levels of both liver and right kidney cortex were obtained on the same longitudinal sonographic plane.

Muscle ultrasound, performed by an eleven linear probe at the level of the biceps muscle of the left superior arm, is a feasible and reliable technique to visualize altered muscle tissue, as in case of ImTG, since it is non-invasive and provides results in real-time. Infiltration of fat and fibrous tissue increases muscle echo intensity, i.e., the muscles appears whiter at the ultrasound image. To describe muscle echo intensity, Heckmatt et al. proposed a four-grade visual scale, where grade I represented normal muscle and grade IV severely increased muscle echo intensity with total loss of bone echo (we chose biceps versus humerus) [25]. Brightness levels of the liver and biceps were calculated three times directly from the frozen images.

The common carotid, the carotid bulb and the near and far wall segments of the internal carotid were scanned bilaterally with a 7.5-MHz linear array transducer, according to the consensus statement from the American Society of Echocardiography Carotid Intima-Media Thickness Task Force, endorsed by the Society for Vascular Medicine [26]. Subjects were examined in the supine position with the head turned 45° contra-lateral to the side of scanning. Images were obtained in longitudinal sections, with a single lateral angle of isonation, optimizing the image for the far wall. IMT was defined as the distance between the lumen-intima and the media-adventitia ultrasound interfaces. Measurements were performed off-line and consisted of six manual measurements at equal distances along 1 cm on the far wall of the common carotid. Left and right IMT were averaged.

### Blood pressure measurements

Systolic/Diastolic Arterial Pressure (SBP, DBP) was the average of three consecutive readings taken by the physician during the day, during usual practice hours, after allowing the subjects to rest for five minutes in the sitting position. Patients on antihypertensive drugs maintained a balanced therapeutic regimen throughout the study.

### Laboratory data

Serum TriGlycerides (TG), HDL, basal insulin, ALT, γ-GT, Alkaline Posphatase (AP), CholinEsterase (CHE), glycemia, ferritin, fibrinogen and insulin were performed by in-house standard procedures. Hs-CRP values were determined by the ELISA test, with reference values ranging between 0.3 and 8.6 mg/L in healthy men and between 0.2 and 9.1 mg/L in healthy women (BioCheck, Inc CA, USA). HSP-70 was determined using a high sensitivity EIA kit by Enzo Life Sciences International, Inc., PA, USA as described in detail in a previous report [24].

### Bead-based assay

Human eotaxin singleplex was performed according to Bio-Rad systems protocol (Bio-Rad Lab., Inc., Hercules, CA, USA) as reported elsewhere [27], [28]. Sera samples were diluted four times with suitable buffer. Initially, the 96-well filter bottom plate was pre-wet. Fifty microliters of diluted microparticle solution and 50 µl of sample were added to each well in duplicate. Thereafter, the plate was incubated for 1 h and washed three times with wash buffer. Afterwards, 25 µl of diluted biotin antibody were added to each well and incubated for 1 h. The plate was then washed as described above and 50 µl of diluted Streptavidin-PE were added to each well and the plate incubated for 10 min. All incubations were performed at room temperature on an orbital shaker set at 15 g. Finally, the plate was washed again with 100 µl of wash buffer. The median relative fluorescence units were measured using the Luminex 200 analyzer (Luminex, Austin, TX, USA). Eotaxin concentrations were calculated using a standard curve. Control range used for eotaxin id was 19.4±7.2 pg/mL.

The coefficient of variation, calculated by SD/mean x 100 for the intra-assay and inter-assay was <10% and <12%, respectively.

To establish whether eotaxin levels correlated to the other cytokines present in the Human Cytokine 27-Plex Panel (Bio-Rad Lab., Inc., Hercules, CA, USA), i.e., IL-1β, IL-1ra, IL-2, IL-4, IL-5, IL-6, IL-7, IL-8, IL-9, IL-10, IL-12 (p70), IL-13, IL-15, IL-17, eotaxin (CCL11), basic FGF, G-CSF, GMCSF, IFN-g, IP-10 (CXCL10), MCP-1 (CCL2), MIP-1a (CCL3), MIP-1b (CCL4), PDGF-bb, RANTES (CCL5), TNF- α and VEGF, we evaluated, by means of the procedure reported above, the following parameters in relation to the respective control ranges: for IL-1b: 0.37±0.21 pg/mL, for IL-6: 4.01±2.58 pg/mL, for TNF-α: 5.76±3.44 pg/mL, for VEGF: 0.44±0.21 pg/mL and for PDGF-BB: 73.53±48.95 pg/mL.

### Statistics

Age, BMI, WHR, SAT, VAT, ALT, AP, γ-GT, HOMA, SBP, DBP, common carotid IMT, TG, CRP, fibrinogen, ferritin, eotaxin, TNF-α, IL-6, Il-1β, VEGF, PDGF-BB were not normally distributed when analyzed by the Shapiro-Wilk (S-W) test, p<0.05, and were expressed as median plus 25–75 inter-quartile range (IQR). Data for WC, CHE HDL cholesterol, SLD, derived from a normally distributed population (S-W, p>0.05), are shown as mean plus SD. At univariate analysis, to assess the independent effect of a quantitative variable on the prediction of another variable, the linear regression analysis (least squares) was used, evaluating the coefficient with its standard error, 95% confidence intervals (CI) and t (t-stat or t-values). A t-stat greater than 1.96 with a significance less than 0.05 indicates that the independent variable is a significant predictor of the dependent variable within and beyond the sample. The scatter plots display the correlation. At multivariate analysis, the multiple regression (Backward Stepwise Selection) was adopted, firstly entering all variables if p = 0.05 in the model, and then removing if p = 0.1 the non-significant variables sequentially, with a maximum number of 15 steps. To avoid multi-collinearity, i.e., situations in which the predictors are correlated with each other to some degree, the variance inflation factor and tolerance were set at>10 and <0.1, respectively. Similarly, to assess which variables contribute more or less to the regression equation, the magnitude of standardized coefficient beta (β) was calculated. Finally the zero order correlation coefficients, indicating the simple correlation coefficients, were evaluated.

To evaluate the intra/inter-observer variability of the measurements, the mean difference in the measurements of the observers was first calculated. Next, the concordance correlation coefficient (ρ_c_), which measures precision and accuracy, was adopted to evaluate the degree of pair observations at US, with values>0.8 considered as indicators of good reliability. MedCalc, version 12–7 (MedCalc Software, Broekstraat 52, 9030 Mariakerke, Belgium) and SyStat 13 (Cranes Software International, Bangalore, India) were the statistical packages employed.

## Results

The demographic assessment, anthropometric measures, laboratory data and instrumental findings of our study population are shown in Table 1.

**Table 1:** Characteristics of Obese Patients (n 80).

Parameter	Mean+/−SD or median plus (25–75 IQR)	Parameter	Mean+/−SD or median plus (25–75 IQR)
Age (years)	46 (34–53)	HDL Females mg/dL	49.3+/−15
Gender M/F	36/44	HDL Males mg/dL	42.7+/−9
Obesity Degree I/II/III (n)	18/26/46	TG mg/dL	123.5 (83.5–188)
BMI	42.3 (38.1–46.8)	CRP mg/mL	0.56 (0.27–1.3)
WC Females (cm)	118.9+/−12.5	Fibrinogen g/L	295.5 (256–357.5)
WC Males (cm)	129.3+/−14	Ferritin Females ng/mL	41.5 (20–69)
WHR Females	0.95 (0.93–0.97	Ferritin Males ng/mL	167.5 (85–234)
WHR Males	0.98 (0.96–1)	SLD cm	11.3+/−1.5
HS Grade 1/2/3 at US (n)	22/48/10	IMT (mm)	0.09 (0.07–011)
SAT (cm)	2.6 (2.1–3.1)	Eotaxin pg/mL	24.6 (6.17–77.5)
VAT (cm)	7.5 (6–9.4)	TNF- α pg/mL	41.8 (8.1–112.7)
ImTG Score I/II/III/IV at US (n)	11/23/30/16	IL-6 pg/mL	5.7 (2.3–17.5)
ALT (U/L)	28 (21.5–29)	IL-1β pg/mL	0.44 (0.2–1.08)
CHE (U/L)	9671.4+/−1882.2	VEGF pg/mL	0.2 (0.2–1.14)
AP (U/L)	73 (61–91)	PDGF-BB pg/mL	468.3 (163.8–1306.3)
γ-GT(U/L)	25 (16.5–42.5)	HSP-70 ng/mL	0.24 (0.10–0.67)
HOMA	2.78 (1.85–4.18)	Fasting insulin µU/mL	10.9 (7.5–15.8)
SBP mm Hg	130 (120–140)	DBP mm Hg	80 (80–90)

Values expressed as means plus/minus standard deviation or median plus 25–75 interquartile range, according to their distribution. Abbreviations: Waist Circumference WC, Body Mass Index BMI, Waist to Hip ratio WHR, Subcutaneous Adipose Tissue SAT, Visceral Adipose Tissue VAT, High Density Lipoprotein-cholesterol HDL, TriGlycerides TG, C Reactive Protein CRP, Spleen Longitudinal Diameter SLD, Tumor Necrosis Factor alpha TNF-α, Interleukin-6 IL-6, Interleukin-1β IL-1β, UltraSound US, ALanine aminoTransferase ALT, CHolinEsterase CHE, Alkaline Phosphatase AP, Gamma-Glutamyl Transglutaminase γ-GT, Vascular Endothelial Growth Factor VEGF, Platelet-Derived Growth Factor-BB PDGF-BB, Heat Shock Protein-70 HSP-70, High Density Lipoprotein cholesterol HDL, HOmeostatic Metabolic Assessment HOMA, Systolic Blood Pressure SPP, Diastolic Blood Pressure DBP, Intima-Media Thickness IMT.

It is noteworthy to stress that the patients in our series presented high grade obesity, i.e., grade II and III, with very large waists, and therefore with evident visceral adiposity.

When analyzing the laboratory data, the patients selected were characterized by normal or slightly elevated liver enzymes. At the instrumental analysis, most of our obese patients showed a light/moderate grade of HS at US, without significantly enlarged spleen. The moderately increased IMT value clearly evidenced an asymptomatic, early stage atherosclerosis in this population, in agreement with the non advanced median age of the participants. Furthermore, data concerning blood pressure, revealed that only mild hypertension was present among our patients.

The values of circulating eotaxin were increased when compared to our reference values.

### Correlations: univariate analysis

The predictions between serum eotaxin concentrations and anthropometric data, inflammation markers, liver parameters (including enzymes and HS grade), angiogenetic factors and cardiovascular risk factors are represented in Table 2. Of some interest were the relationships between eotaxin concentrations and the following specific immune regulators, i.e., TNF- α, IL-6, IL-1β on the one hand, and between eotaxin and angiogenetic factors, i.e., VEGF as well as PDGF-BB on the other. Among the metabolic parameters, anthropometric data and US features of excess body weight, a significant prediction was only found comparing eotaxin to HOMA. IMT was well predicted by eotaxin. When analyzing other cardiovascular risk factors associated with serum eotaxin, blood pressure determinations were non significant, as was HS grade. Furthermore, HSP-70 was not predicted by seum eotaxin.

**Table 2:** Report of Predictions Using Eotaxin As Dependent Variable.

	Coefficient	Std. Error	95% CI	t	P
Eotaxin/WC	−0.001	0.018	−0.038 to 0.034	−0.11	0.91
Eotaxin/BMI	0.0005	0.010	−0.02 to 0.02	0.005	0.99
Eotaxin/WHR	0.0001	0.0006	−0.0003 to 0.0002	1.94	0.06
Eotaxin/SAT	−16.06	12.79	−41.53 to 9.40	−1.25	0.2
Eotaxin/VAT	−1.68	3.9	−9.45 to 6.09	−0.43	0.66
Eotaxin/Fibrinogen	0.05	0.09	−0.13 to 0.24	0.56	0.57
Eotaxin/CRP	−1.12	4.58	−10.31 to 7.92	−0.26	0.79
Eotaxin/SLD	−1.62	6.38	−14.32 to 11.07	−0.25	0.79
Eotaxin/Ferritin	0.038	0.073	−0.10 to 0.18	0.52	0.6
Eotaxin/TNF-α	0.30	0.076	0.14 to 0.45	3.9	**0.0002**
Eotaxin/IL-6	1.59	0.48	0.62 to 2.5	3.3	**0.0016**
Eotaxin/IL-1β	25.86	7.11	11.70 to 40.02	3.63	**0.0005**
Eotaxin/US	−10.91	15.38	−41.5 to 19.71	0.70	0.48
Eotaxin/ALT	−0.01	0.02	−0.054 to 0.02	−0.64	0.52
Eotaxin/CHE	0.0017	0.0052	−0.012 to 0.0087	−0.33	0.73
Eotaxin/AP	−0.18	0.37	−0.93 to 0.56	−0.5	0.61
Eotaxin/*γ-GT*	0.37	0.48	−0.59 to 1.34	0.77	0.44
Eotaxin/VEGF	3.11	1.23	0.65 to 5.57	2.5	**0.013**
Eotaxin/PDGF-BB	0.015	0.006	0.0031 to 0.028	2.49	**0.015**
Eotaxin/HSP-70	4.74	7.95	−11.09 to 20.58	0.59	0.55
Eotaxin/HDL	−0.006	0.015	−0.03 to 0.02	−0.41	0.67
Eotaxin/HOMA	5.89	2.45	1.01 to 10.77	2.4	**0.018**
Eotaxin/Triglycerides	−0.031	0.12	−0.26 to 0.2	−0.27	0.78
Eotaxin/SBP	0.025	0.68	−1.33 to 1.38	0.036	0.97
Eotaxin/DBP	0.048	1.19	−2.33 to 2.43	0.04	0.96
Eotaxin/IMT	705.15	281.69	144.34 to 1265.95	2.50	0.014

Linear Regression with Coefficients and their Standard Error, 95% Confidence Intervals, t values and significance level expressed as P. Abbreviations: Waist Circumference WC, Body Mass Index BMI, Waist to Hip ratio WHR, Subcutaneous Adipose Tissue SAT, Visceral Adipose Tissue VAT, C Reactive Protein CRP, Spleen Longitudinal Diameter SLD, Tumor Necrosis Factor alpha TNF-α, Interleukin-6 IL-6, Interleukin-1β IL-1β, UltraSound US, ALanine aminoTransferase ALT, CHolinEsterase CHE, Alkaline Phosphatase AP, Gamma-Glutamyl Transglutaminase γ-GT, Vascular Endothelial Growth Factor VEGF, Platelet-Derived Growth Factor-BB PDGF-BB, Heat Shock Protein-70 HSP-70, High Density Lipoprotein HDL, HOmeostatic Metabolic Assessment HOMA, Systolic Blood Pressure SPP, Diastolic Blood Pressure DBP, Intima-Media Thickness IMT.

Correlating indices of fat storage at US (SAT, VAT, HS and ImTG), ImTG score was well predicted by HS grade (coefficient  = 0.51, standard error  = 0.17, 95% CI  = 0.17 to 0.85, t = 2.1, P = 0.003) and VAT (coefficient  = 0. 12, standard error  = 0.4, 95% CI = 0.04 to 0.20, t = 3.1, P = 0.0026).

Liver laboratory test such as ALT, γ-GT, AP values were not predicted by eotaxin levels in our series (Table 2). Interestingly, HS grade was predicted by ALT values (coefficient  = 6.8, standard error = 2.9, 95% CI = 0.87 to 12.8, t = 2.28, P = 0.025) and γ-GT values (coefficient  = 10.65, standard error  = 3.7, 95% CI = 3.27 to 18, t = 2. 87, P = 0.005). Finally, HOMA predicted the grade of HS severity at US (coefficient  = 0.056, standard error  = 0.015, 95% CI = 0.025 to 0.088, t = 3.6, P = 0.0005), resulting to be one of the strongest relationships.

**Figure 1:**
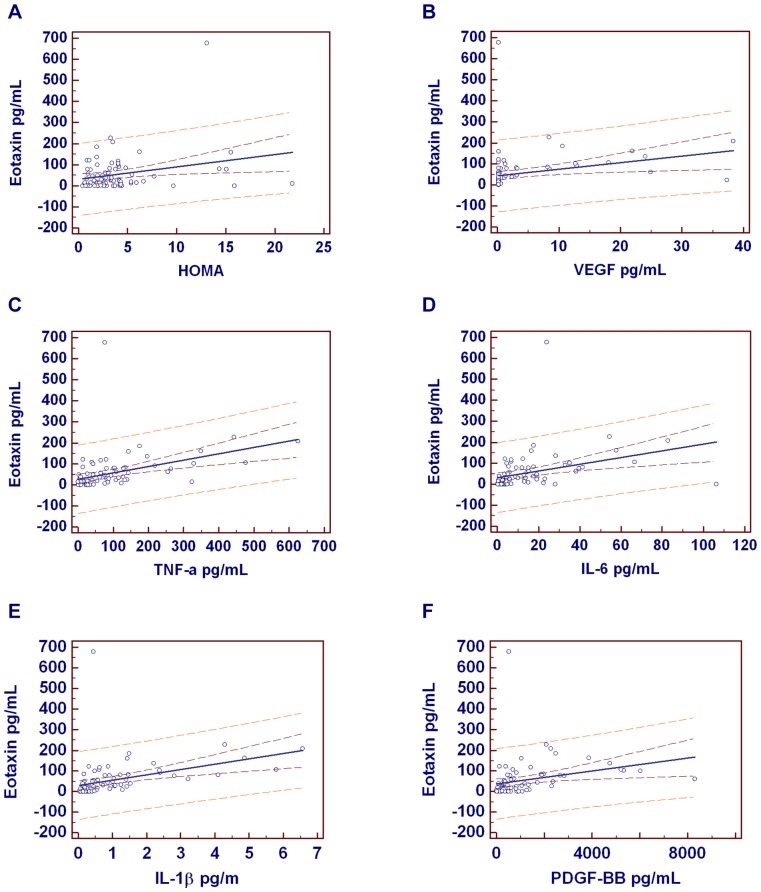
Graphics of significant predictions between eotaxin and other independent variables. Eotaxin was well predicted by HOMA (r = 0.26, P = 0.019), VEGF (r = 0.27, P = 0.013), TNF-α (r = 0.405, P = 0.0002), IL-6 (r = 0.35, P = 0.0016), IL-1β (r = 0.38, P = 0.0005), PDGF-BB (r = 0.27, P = 0.015). Correlation coefficient r. The regression line is evidenced in blue; the 95% confidence in dark red and the 95% prediction in light red. Dependent Y =  Eotaxin.

The most important correlations are shown in Figure 1 (A-F).

### Correlations: multivariate analysis

At the multiple regression analysis, using the stepwise method, two independent variables contextually predicted the dependent variable IMT, i.e., serum eotaxin levels and HS grade at US (beta 0.30, P = 0 0.005 and beta 0.32, P = 0. 0038, respectively). The remaining variables not included in the model were HDL, TG, HOMA and CRP.

The zero order correlation coefficients between IMT and eotaxin, HDL, TG, HOMA CRP and HS at US were 0.27, −0.08, 0.07, 0.18, −0.045, and 0.28, respectively.

Finally, at the multiple regression, forcing the stepwise method, among ALT, AST, γ-GT, AP, CHE and SLD, only spleen volume was retained, predicting HS at US (coefficient  = 0.1815, standard error  = 0.03767, beta = 0.48. P = <0.0001).

### Agreement

The intra/inter-observational variability of UltraSound estimations was not significant, the mean difference being 1.9, 2.9, 2.4, 3.1, 2.4 and 3.9%, and 2.3, 3.1, 3. 9, 3.1, 2.6 and 3.4% for the HS, ImTG, SAT, VAT, SLD and common carotid IMT, respectively, with a ρ_c_ of 0.91.

## Discussion

The key finding of this research can be summarized as follows: Firstly, a clear relationship exists between serum concentrations of eotaxin and TNF α, IL-6, IL-1β. These cytokines have marked effects on numerous cell types which, in turn, secrete a variety of inflammatory mediators, creating complex networks of interactions and leading to multiple inflammatory cascades. Secondly, serum concentrations of eotaxin were significantly related to the serum levels of angiogenetic factors, i.e., VEGF as well as PDGF-BB. The former and latter findings confirm the body of pertinent knowledge that an inflammatory/immune interplay could be evidenced in the induction of atherosclerosis, but also in the maintenance of this process by determining impaired angiogenesis and/or endothelial cell dysfunction. In fact, IL-1 β and TNFα have potent effects on vessel wall components—particularly on endothelial cells, and may influence the mechanisms involved in vessel occlusion and repair [29].

As determined by multivariate analysis, the most important factors in predicting patients with early atherosclerosis were both eotaxin levels and the severity of HS at US, even if we were unable to show any association between these two variables, which likely contribute, in an independent fashion, to determine or worsen the atheromatosus process.

Among the metabolic parameters, a significant prediction was found comparing eotaxin to IR, as detected by HOMA, but not to the other two classic CAD risks, i.e., HDL, TG. Of relative interest is the association, detected only at univariate analysis, of HS with ALT and *γ-GT*, although two limitations in its interpretation must be acknowledged: the small size of the cohort studied and the values of liver enzymes not exceeding twice the normal values). Discussing possible mechanisms and explanations for these findings, we hypothesize that eotaxin is released by the smooth muscle cells present in the atheromatous vessels only by activated cells, after a stimulus by either TNF-α or IL-1β. This interpretation is supported by Ghaffar et al. [30], whose study demonstrated increased eotaxin expression in smooth muscle cultures after treatment with both the aforementioned cytokines. That IR (evaluated by HOMA), the main mechanism underlying HS (confirmed also by the findings of this research) and which plays a key role in linking HS to CVD [21,24], could mediate the increased release of eotaxin from competent cells, surely cannot be inferred by this work, although this hypothesis is suggestive. In any case, the relationship between IR and eotaxin could be based on the GH/IGF axis, which is altered in NAFLD [31]. This hypothesis finds support in a recent study showing that IGFBP-3 inhibits TNF-α, CRP and high glucose-induced NF-κB activity in human aortic endothelial cells, and subsequently suppresses monocyte adhesion in the same cells through the IGFBP-3 receptor [32].

Very scarce data was found in relation to the link between eotaxin and IMT when comparing our results with relevant findings from other reports found in the literature by search sources (MEDLINE/SCOPUS) during the last decade, in which plasma eotaxin levels accurately identified individuals with clinically significant atherosclerotic heart disease [7] and were associated with the presence and extend of angiographic coronary artery disease [8]. The results of the present study on obese patients suffering from NAFLD allows us to confirm the main role of eotaxin in the atherosclerotic process (likely early stage), a process that is more and more reckoned to be based on immune-allergic mechanisms. As to the limitations of the present study, we first of all should pinpoint the reduced sample size of the selected population. A further drawback is that we did not perform liver biopsies because of technical (repeated passages due to the amount of subcutaneous adipose tissue could increase procedure risks) and ethical issues (lack of significant hepatic cytolysis). Neither was adiposity evaluated by MRI, nor was arterial stiffness detected by means of pulse wave velocity, both known to be more precise methods. It should, however, be emphasized that the major advantage of IMT is that it is completely non-invasive and can be repeatedly performed in follow-up studies. It is also relatively inexpensive, and the technology widely available. Increased IMT has been shown to consistently predict future vascular events [33]. IMT is an alternative to coronary calcium scans and does not expose patients to any radiation. The NIH, as reported in their public access, also accepts IMT (but not coronary calcium scanning, which utilization needs further studies) to follow patients for atherosclerotic disease progression [34]. Furthermore, compared to other more sophisticated methods, it has proven to be very reliable [35].

Given the experience accumulated in experimental settings and in human disease showing that the systemic inflammatory response is triggered by pro-inflammatory cytokines, crucial future research in obese patients should be directed towards the study of the interplay between cytokine/chemokines and other angiogenetic factors; in fact, basic Fibroblast Growth Factor (b-FGF), which activates monocytes to promote the endothelial cell proliferation involved in plaque development [36], seems to be one of the most promising lines of research.

## Conclusion

The atherosclerotic process is not fully understood. Canonical data link hyperlipidemia, hypertension and cigarette smoking—which together increase risk manifold—to atherogenesis. Obese individuals with NAFLD are a properly selected population to study this illness in an early phase. IMT can be reduced by lifestyle modifications and therapeutic interventions. However, only recently have we appreciated that inflammation couples dyslipidaemia to atheroma formation. Leukocyte recruitment and expression of pro-inflammatory cytokines characterize early atherogenesis, which is initiated by inflammatory processes in the endothelial cells of the vessel wall in response LDL particles [37]. Moreover, inflammatory pathways promote thrombosis, a late and dreaded complication of atherosclerosis, which is responsible for myocardial infarctions and most strokes. The clinical implications of this work, summarized in a straightforward and circumspect manner, could consist in identifying the triggers for inflammation, and unraveling the details of the inflammatory pathways may eventually furnish new therapeutic targets.

## References

[1] Berliner JA, Navab M, Fogelman AM, Frank JS, Demer LL, et al (1995) Atherosclerosis: basic mechanisms. Oxidation, inflammation, and genetics. Circulation 91: 2488–2496. 7729036 10.1161/01.cir.91.9.2488

[2] Nordestgaard BG, Zacho J (2006) Lipids, atherosclerosis and CVD risk: is CRP an innocent bystander? Nutr Metab Cardiovasc Dis 19: 521–524. 10.1016/j.numecd.2009.07.005 19695857

[3] Thompson RC, Allam AH, Lombardi GP, Wann LS, Sutherland ML, et al (2013) Atherosclerosis across 4000 years of human history: the Horus study of four ancient populations. Lancet 381: 1211–1222. 23489753 10.1016/S0140-6736(13)60598-X

[4] Xu JM, Shi GP (2012) Emerging role of mast cells and macrophages in cardiovascular and metabolic diseases. Endocr Rev 33: 71–108. 22240242 10.1210/er.2011-0013 PMC3365842

[5] Wang J, Cheng X, Xiang MX, Alanne-Kinnunen M, Wang JA, et al (2011) IgE stimulates human and mouse arterial cell apoptosis and cytokine expression and promotes atherogenesis in Apoe−/− mice. J Clin Invest 121: 3564–3577. 21821913 10.1172/JCI46028 PMC3163955

[6] Rothenbacher D, Müller-Scholze S, Herder C, Koenig W, Kolb H (2006) Differential expression of chemokines, risk of stable coronary heart disease, and correlation with established cardiovascular risk markers. Arterioscler Thromb Vasc Biol 26: 194–199. 16239601 10.1161/01.ATV.0000191633.52585.14

[7] Emanuele E, Falcone C, D'Angelo A, Minoretti P, Buzzi MP, et al (2006) Association of plasma eotaxin levels with the presence and extent of angiographic coronary artery disease. Atherosclerosis 186: 140–145. 16084515 10.1016/j.atherosclerosis.2005.07.002

[8] Ardigo D, Assimes TL, Fortmann SP, Go AS, Hlatky M, et al (2007) Circulating chemokines accurately identify individuals with clinically significant atherosclerotic heart disease. Physiol Genomics 31: 402–409. 17698927 10.1152/physiolgenomics.00104.2007

[9] Wang L, Shah PK, Wang W, Song L, Yang M, et al (2013) Tenascin-C deficiency in apo E−/− mouse increases eotaxin levels: implications for atherosclerosis. Atherosclerosis 227: 267–274. 23433402 10.1016/j.atherosclerosis.2013.01.039 PMC3621962

[10] Haley KJ, Lilly CM, Yang JH, Feng Y, Kennedy SP, et al (2000) Turi TG, Thompson JF, Sukhova GH, Libby P, Lee RT. Overexpression of eotaxin and the CCR3 receptor in human atherosclerosis: using genomic technology to identify a potential novel pathway of vascular inflammation. Circulation 102: 2185–219. 11056090 10.1161/01.cir.102.18.2185

[11] Duval C, Thissen U, Keshtkar S, Accart B, Stienstra R, et al (2010) Adipose tissue dysfunction signals progression of hepatic steatosis towards nonalcoholic steatohepatitis in C57BL/6 mice. Diabetes 59: 3181–3191. 20858684 10.2337/db10-0224 PMC2992781

[12] Tarantino G, Finelli C (2013) What about non-alcoholic fatty liver disease as a new criterion to define metabolic syndrome? World J Gastroenterol 19: 3375–3384. 23801829 10.3748/wjg.v19.i22.3375 PMC3683675

[13] Tarantino G, Caputi A (2011) NKs, insulin resistance and inflammation: A possible link between NAFLD and coronary artery disease. World J Gastroenterol 17: 3785–3794. 21987620 10.3748/wjg.v17.i33.3785 PMC3181439

[14] Hamaguchi M, Kojima T, Itoh Y, Harano Y, Fujii K, et al (2007) The severity of ultrasonographic findings in nonalcoholic fatty liver disease reflects the metabolic syndrome and visceral fat accumulation. Am J Gastroenterol 102: 2708–2715. 17894848 10.1111/j.1572-0241.2007.01526.x

[15] Rabøl R, Svendsen PF, Skovbro M, Boushel R, Haugaard SB, et al (2009) Reduced skeletal muscle mitochondrial respiration and improved glucose metabolism in nondiabetic obese women during a very low calorie dietary intervention leading to rapid weight loss. Metabolism 58: 1145–1152. 19454354 10.1016/j.metabol.2009.03.014

[16] Shimabukuro M, Kozuka C, Taira S, Yabiku K, Dagvasumberel M, et al (2013) Ectopic fat deposition and global cardiometabolic risk: new paradigm in cardiovascular medicine. J Med Invest 60: 1–14. 23614905 10.2152/jmi.60.1

[17] Pillen S, Arts IMP, Zwarts MJ (2008) Muscle ultrasound in neuromuscular disorder. Muscle nerve 37: 679–693. 18506712 10.1002/mus.21015

[18] de Koning L, Merchant AT, Pogue J, Anand SS (2007) Waist circumference and waist-to-hip ratio as predictors of cardiovascular events: meta-regression analysis of prospective studies. Eur Heart J 28: 850–856. 17403720 10.1093/eurheartj/ehm026

[19] Barter P (2011) HDL-C: role as a risk modifier. Atheroscler Suppl 12: 267–270. 10.1016/S1567-5688(11)70885-6 22152280

[20] Fathi R, Marwick TH (2001) Noninvasive tests of vascular function and structure: why and how to perform them. Am Heart J 141: 694–703. 11320355 10.1067/mhj.2001.114972

[21] Tarantino G, Colicchio P, Conca P, Finelli C, Di Minno MN, et al (2009) Young adult obese subjects with and without insulin resistance: what is the role of chronic inflammation and how to weight it non-invasively? J inflamm (London) 6: 6. 19291292 10.1186/1476-9255-6-6 PMC2663560

[22] Heinonen SE, Kivelä AM, Huusko J, Dijkstra MH, Gurzeler E, et al (2013) The effects of VEGF-A on atherosclerosis, lipoprotein profile, and lipoprotein lipase in hyperlipidaemic mouse models. Cardiovasc Res 99: 716–723. 23756254 10.1093/cvr/cvt148

[23] Qi YX, Jiang J, Jiang XH, Wang XD, Ji SY, et al (2011) PDGF-BB and TGF-{beta}1 on cross-talk between endothelial and smooth muscle cells in vascular remodeling induced by low shear stress. Proc Natl Acad Sci U S A 108: 1908–1913. 21245329 10.1073/pnas.1019219108 PMC3033274

[24] Tarantino G, Finelli C, Colao A, Capone D, Tarantino M, et al (2012) Are hepatic steatosis and carotid intima media thickness associated in obese patients with normal or slightly elevated gamma-glutamyl-transferase? J Transl Med 10: 50. 22424154 10.1186/1479-5876-10-50 PMC3342159

[25] Heckmatt JZ, Leeman S, Dubowits V (1982) Ultrasound imaging in the diagnosis of muscle disease. J Pediatr 101: 656–660. 7131136 10.1016/s0022-3476(82)80286-2

[26] Stein JH, Korcarz CE, Hurst RT, Lonn E, Kendall CB, et al (2008) Use of carotid ultrasound to identify subclinical vascular disease and evaluate cardiovascular disease risk: a consensus statement from the American Society of Echocardiography Carotid Intima-Media Thickness Task Force. Endorsed by the Society for Vascular Medicine. J Am Soc Echocardiogr 21: 93–111. 18261694 10.1016/j.echo.2007.11.011

[27] Arellano-Garcia ME, Hu S, Wang J, Henson B, Zhou H, et al (2008) Chia D, Wong DT. Multiplexed immunobead-based assay for detection of oral cancer protein biomarkers in saliva. Oral Dis 14: 705–12. 19193200 10.1111/j.1601-0825.2008.01488.x PMC2675698

[28] Lédée N, Munaut C, Sérazin V, Perrier d'Hauterive S, Lombardelli L, et al (2010) Performance evaluation of microbead and ELISA assays for follicular G-CSF: a non-invasive biomarker of oocyte developmental competence for embryo implantation. J Reprod Immunol 86: 126–132. 20594599 10.1016/j.jri.2010.05.003

[29] Bhagat K, Vallance P (1999) Effects of cytokines on nitric oxide pathways in human vasculature. Curr Opin Nephrol Hypertens 8: 89–96. 9914865 10.1097/00041552-199901000-00014

[30] Ghaffar O, Hamid Q, Renzi PM, Allakhverdi Z, Molet S, et al (1999) Constitutive and cytokine-stimulated expression of eotaxin by human airway smooth muscle cells. Am J Respir Crit Care Med 159: 1933–1942. 10351942 10.1164/ajrccm.159.6.9805039

[31] Savastano S, Di Somma C, Pizza G, De Rosa A, Nedi V, et al (2011) Liver-spleen axis, insulin-like growth factor-(IGF)-I axis and fat mass in overweight/obese females. J Transl Med 9: 136. 21846339 10.1186/1479-5876-9-136 PMC3177905

[32] Mohanraj L, Kim HS, Li W, Cai Q, Kim KE, et al (2013) IGFBP-3 inhibits cytokine-induced insulin resistance and early manifestations of atherosclerosis. PLoS One 8: e55084. 23383064 10.1371/journal.pone.0055084 PMC3557269

[33] Jeevarethinam A, Venuraju S, Weymouth M, Atwal S, Lahiri A (2014) Carotid Intimal Thickness and Plaque Predict Prevalence and Severity of Coronary Atherosclerosis: A Pilot Study. Angiology Feb 26. 10.1177/0003319714522849 24576983

[34] Nathan DW, Lopez VA, Matthew Allison MS, Robert C, Detrano RC, et al (2011) Abdominal Aortic Calcium and Multi-Site Atherosclerosis: TheMultiethnic Study of Atherosclerosis. Atherosclerosis 214: 436–441. 21035803 10.1016/j.atherosclerosis.2010.09.011 PMC3040451

[35] Schroeder B, Francis G, Leipsic J, Heilbron B, John Mancini GB, et al (2013) Early atherosclerosis detection in asymptomatic patients: a comparison of carotid ultrasound, coronary artery calcium score, and coronary computed tomography angiography. Can J Cardiol 29: 1687–94. 24267808 10.1016/j.cjca.2013.10.003

[36] Pakala R, Watanabe T, Benedict CR (2002) Induction of endothelial cell proliferation by angiogenic factors released by activated monocytes. Cardiovasc Radiat Med 2002 3: 95–101. 10.1016/s1522-1865(02)00159-2 12699839

[37] Williams KJ, Tabas I (1995) The Response-to-Retention Hypothesis of Early Atherogenesis. Arterioscler Thromb Vasc Biol 15: 551–61. 7749869 10.1161/01.atv.15.5.551 PMC2924812

